# Community Social Capital and All-cause Mortality in Japan: Findings From the Adachi Cohort Study

**DOI:** 10.2188/jea.JE20240277

**Published:** 2025-06-05

**Authors:** Hiroshi Murayama, Mika Sugiyama, Hiroki Inagaki, Ayako Edahiro, Fumiko Miyamae, Chiaki Ura, Keiko Motokawa, Tsuyoshi Okamura, Shuichi Awata

**Affiliations:** 1 Research Team for Social Participation and Healthy Aging, Tokyo Metropolitan Institute for Geriatrics and Gerontology, Tokyo, Japan; 2 Research Team for Promoting Independence and Mental Health, Tokyo Metropolitan Institute for Geriatrics and Gerontology, Tokyo, Japan; 3 Integrated Research Initiative for Living Well with Dementia, Tokyo Metropolitan Institute for Geriatrics and Gerontology, Tokyo, Japan

**Keywords:** social capital, mortality, neighborhood cohesion, neighborhood network, Japan

## Abstract

**Background:**

Community social capital is associated with various health outcomes; however, its impact on mortality is not fully understood, particularly in non-Western settings. This study examined the association between community-level social capital and all-cause mortality among community-dwelling older Japanese adults.

**Methods:**

The baseline data were obtained from a 2015 questionnaire survey for all 132,005 residents aged ≥65 years without long-term care insurance certification in Adachi Ward (consisting of 262 small districts) of the Tokyo metropolitan area. We measured two aspects of social capital: neighborhood cohesion as cognitive social capital and neighborhood network as structural social capital. For district-level social capital, we aggregated the individual responses of neighborhood cohesion and neighborhood network in each district.

**Results:**

A total of 75,338 were analyzed. A multilevel survival analysis with an average follow-up of 1,656 days showed that higher district-level neighborhood cohesion was associated with a lower risk of all-cause mortality in men (hazard ratio 0.92; 95% confidence interval [CI], 0.84–0.99 for the highest quintile and 0.91; 95% CI, 0.82–0.99 for the second, compared to the lowest), but not in women. This association was more pronounced in men aged 65–74 years.

**Conclusion:**

This study provides valuable insights from the Asian population. Men, who typically have fewer social networks and support systems than women, could receive more benefits from residing in a cohesive community, which may contribute to their longevity. These findings support public health strategies that bolster community social capital as a means of archiving longevity among older men, underscoring the importance of social integration in aging societies.

## INTRODUCTION

The association between social capital and health outcomes has been a focal point of study within the field of social epidemiology. Social capital is an aspect of the social features of a community that may be modifiable and can be defined as the resources accessed by individuals as a result of their membership in a community.^1^ Although all individuals in the same specific context (eg, districts, workplaces, or schools) have equal social exposure, related effects on health may vary between individuals. The concept of social capital can be broken down into several forms and dimensions. A common distinction in research on social capital is between the structural and cognitive dimensions.^1^ The cognitive dimension reflects subjective attitudes, perceptions, and cognitions about the group to which people belong, such as trust in others and norms of reciprocity. The structural dimension includes externally observable aspects of social organization and is characterized by behavioral manifestations of network connections or civic engagement.

The existing literature shows methodological diversity and presents mixed findings, ranging from cross-sectional to longitudinal studies that employ diverse measures of social capital. Ehsan et al reviewed previous systematic reviews on social capital and health and found that, although community social capital was often protective against a poorer health status, there was a lack of clear evidence regarding how different elements of social capital influence the health consequences of diverse groups.^2^

Regarding all-cause mortality, in addition to the limited number of previous studies, the relationship between community social capital and mortality remains unclear and controversial.^3^^,^^4^ eTable 1 outlines a literature review of research focused on the relationship between community social capital and all-cause mortality. Prior ecological studies have shown that higher social capital (including both cognitive and structural dimensions) is associated with a lower mortality rate,^5^^,^^6^ while others have shown no such relationship.^7^ Studies using multilevel modelling have also shown conflicting results. The effects of social capital on mortality risk have been reported in the United States,^8^^,^^9^ the United Kingdom,^10^ Sweden,^11^^,^^12^ Brazil,^13^ and South Korea.^14^ For example, a nationwide Swedish study by Sundquist et al showed that low neighborhood-level linking social capital, defined by voting rate (ie, structural dimension), was associated with higher risks of all-cause mortality in the general population aged ≥65 years.^11^ In contrast, a study focused on Chicago, United States conducted by Wen et al found that the density of community social networks (structural social capital) had a detrimental effect on all-cause mortality.^9^ Furthermore, two studies from New Zealand^15^ and Japan^16^ demonstrated no such association. A Japanese study conducted in a prefecture by Inoue et al used data from adults aged 65–84 years and found that community-level social cohesion (ie, cognitive dimension) did not correlate with all-cause mortality.^16^ These variations have led to a complex understanding of the relationship between community social capital and mortality.

This association can be affected by geographical and cultural variability.^4^ Recent studies have suggested that genetic elements may play a role in social capital, underscoring the importance of research in diverse cohorts.^17^ Many previous studies have involved Western populations; thus, more research with non-Western populations (including Asian populations) is warranted. Building social capital in the community is now a priority in public health practice. Therefore, the effect of developing community social capital should be demonstrated to evaluate the practices not only in Western countries but also in non-Western nations. Recognizing these research gaps, especially the need for studies in diverse cultural and contextual settings, our study was conducted in Japan and focused on older adults residing in urban areas. This demographic and geographical specificity addresses a significant gap in the literature by providing insight into the contextual effects of social capital on mortality among older Japanese individuals, a group facing unique societal and health challenges.

Further, important to the current research, sex is another factor affecting the relationship between community social capital and mortality. For example, previous studies have shown that women are more likely to have larger social networks than men.^18^^,^^19^ In addition, life expectancy tends to be longer in women than in men in most countries worldwide.^20^ Therefore, sex is possibly a strong confounder in the relationship between social capital and mortality.

Accordingly, this study examined the association between community social capital and all-cause mortality among community-dwelling older adults. In the analysis, we stratified the participants by sex.

## METHODS

### Study design, participants, and setting

Baseline data were obtained from a questionnaire survey for all community-dwelling individuals aged ≥65 years in July 2015, in Adachi Ward, Tokyo, Japan. Adachi Ward is located in the northeastern part of the Tokyo metropolitan area. As of July 1, 2015, the total population size was 677,531 (339,951 men and 337,580 women), the population density was 12,723.6/km^2^, and the proportion of people aged ≥65 years was 24.3%. Adachi comprises 262 small districts (*chou-chou* in Japanese).

As of April 1, 2015, Adachi had 163,719 residents aged ≥65 years. After excluding people with long-term care insurance certification (including both required support levels 1–2 and care levels 1–5; *n* = 29,327) and those who had died, moved away, or had newly received long-term care insurance certification by the date the questionnaire was dispatched (*n* = 2,387), a self-administered questionnaire was distributed by mail to 132,005 people.

The study protocol was approved by the Ethics Committee of the Tokyo Metropolitan Institute for Geriatrics and Gerontology (approval number: 26-2792). The participants received a detailed written explanation of the study, and all participants provided informed consent prior to engaging in the study. Consent for participation was implied by their returning the completed questionnaire.

### Measures

#### All-cause mortality

We systematically recorded all-cause mortality until April 30, 2020. The observations spanned 57 months after the initial survey. We gathered the dates of death or migration of the participants by consulting local databases synchronized with the Japanese National Vital Statistics system. During this monitoring interval, 9.0% (*n* = 6,806) of the participants died. Those who moved out of Adachi Ward were treated as censored, accounting for 2.8% (*n* = 2,113). On average, each participant was followed for 1,656 days.

#### Individual- and district-level social capital

We adopted two aspects of social capital in this study: cognitive social capital (individual attitudes, perceptions, and cognitions about the group to which they belonged) and structural social capital (behavioral manifestations of network connections or civic engagement).^1^ Cognitive and structural social capital were measured using neighborhood cohesion and networks, respectively. These measurements were not validated. However, previous studies have used these measures to examine the association between social capital and health consequences.^21^^,^^22^

At the individual level, neighborhood cohesion is a measure of unity and mutual support within a community, symbolizing the benefits one can derive from being part of it. It differs from the concepts of social network interaction, support, and social exchange because it evaluates the community’s shared norms and behavioral expectations rather than tangible relationships or direct interactions among individuals.^23^ This study assessed neighborhood cohesion using three items: neighborhood trust (“Do you trust your neighbors?”), neighborhood attachment (“Do you have an attachment to your residential neighborhood?”), and sense of belonging to the neighborhood (“Do you feel that you are a member of your residential neighborhood?”). The possible answers for each item were 1 = “disagree,” 2 = “slightly disagree,” 3 = “slightly agree,” or 4 = “agree.” These three items were aggregated into a single scale (ranging from 3–12). Higher scores indicate higher levels of neighborhood cohesion. Based on the score distribution, we classified the responses as weak (score of 3–8), moderate (9–10), and strong (11–12).

The individual-level neighborhood network was assessed in terms of the density of social relationships with neighbors. We used the following single item: “How is your relation with your neighbors?” Respondents answered: 1 = “I am not friendly with my neighbors at all,” 2 = “I only greet my neighbors,” 3 = “I only make small talk with my neighbors,” or 4 = “I often talk with neighbors about my problems.” Responses were classified as weak (responses 1 and 2), moderate (response 3), or strong (response 4).

Participants were nested within small districts (*n* = 262) in Adachi. Therefore, to obtain district-level social capital indicators, we aggregated individual responses and calculated the proportion of each of the two social capital indicators among the respondents in each district: the proportions of those with moderate and strong neighborhood cohesion (as district-level cognitive social capital) and neighborhood networks (as district-level structural social capital). In the analysis, we divided these proportions into quintiles: Q1 (highest), Q2, Q3, Q4, and Q5 (lowest): ≥69.21%, 66.01–69.20%, 63.53–66.00%, 60.16–63.52%, and ≤60.15% for neighborhood cohesion, and ≥70.20%, 67.12–70.19%, 63.91–67.11%, 60.40–63.90%, and ≤60.39% for neighborhood networks, respectively.

#### Covariates

Sex, age, years of residence in the neighborhood, marital status, household composition, employment status, socioeconomic status (SES), smoking habits, regular exercise habits, body mass index, self-rated health, presence of diagnosed diseases (hypertension, hyperlipidemia, heart disease, stroke, diabetes, and cancer), and depression were included as individual-level covariates. Sex and age data were acquired from the basic resident register of Adachi Ward. The other variables were ascertained using questionnaires.

SES includes years of education and subjective financial stability. Subjective financial stability was measured using a five-point Likert scale and responses were classified into three categories: affluent, normal, and poor. Body mass index was calculated from self-reported height and weight and classified into underweight (<18.5 kg/m^2^), normal weight (18.5–24.9 kg/m^2^), and overweight (≥25.0 kg/m^2^). To assess depression, a previously validated two-question case-finding instrument was used.^24^

Additionally, we used district-level demographic and SES indicators as covariates. For district-level demographic variables, we considered the proportion of people aged ≥65 years and the proportion of people who have lived in their current neighborhood since birth in each district. For district-level SES variables, we considered the proportion of people who graduated from junior high school only and the proportion of people with blue-collar jobs in each district. This information was obtained from the 2010 Japanese Census and the proportion of each district was calculated. To understand the characteristics of the districts, the population size of each district was determined using the same census data.

### Statistical analysis

We fitted the data using a multilevel mixed-effects parametric survival analysis that included district-level random effects (ie, random intercepts) in the model to assess the risk of mortality. We conducted analyses according to sex. The fixed effects results were expressed as hazard ratios (HRs) with 95% confidence intervals (CIs).

As district-level social capital is composed of aggregated individual responses, individual-level social capital may have been a confounding factor in the contextual association between social capital and mortality.^25^ Therefore, individual- and district-level social capital indicators were simultaneously added to the model. To address multicollinearity between social capital variables at the individual and district levels, indicators of social capital at the individual level were centered on the district mean. Social capital indicators were added to the model using the following modeling strategy: i) neighborhood cohesion (model 1), ii) neighborhood networks (model 2), and iii) both neighborhood cohesion and neighborhood networks (model 3). All individual- and district-level covariates were controlled for in each model. In addition, to assess whether the effect of social capital on mortality varied with participant background, we examined the cross-level interaction between district-level social capital and individual-level sociodemographic factors in model 3. When any significant interactions were detected, we performed an analysis stratified by the factors.

Furthermore, we undertook multiple imputations to minimize missing data resulting from item non-response. Specifically, 20 complete datasets were imputed, and the results presented in this paper have been derived from the imputed datasets. All analyses were performed using STATA 17 (StataCorp LLC, College Station, TX, USA).

## RESULTS

In total, 78,917 questionnaires were returned (response rate: 59.8%). We targeted community-dwelling people; thus, after excluding respondents who were not living in their own homes (*n* = 3,559), 75,358 were regarded as valid responses (valid response rate: 57.1%). Participants were nested in 262 districts. However, because two of the 262 districts had fewer respondents than 30 (the number of participants who came from these two districts was 5 and 15, respectively), we excluded them. Consequently, the total analytical sample size was 75,338 across 260 districts. The average number of participants in each district was 289.8 (ranging from 44–763).

Table 1 presents the baseline characteristics of the participants. Men accounted for 45.0% of the sample, and the mean age was 73.7 years. Regarding social capital, 20.8% and 16.0% of respondents had strong neighborhood cohesion and neighborhood networks, respectively. Table 2 lists the characteristics of the districts. The mean population size was 2,626.7 persons, and the mean aging rate was 22.0%. The proportion of districts that reported moderate or strong neighborhood cohesion networks was 63.5%.

**Table 1.** Characteristics of the participants

		Non-imputed	Imputed		
	
		Total *n* = 75,338	Total *n* = 75,338	Men *n* = 33,881	Women *n* = 41,457
Sex	Men	45.0	45.0	—	—
	Missing	0.0			
Age, years		73.7 (6.0)	73.7 (6.0)	73.6 (6.1)	73.8 (5.9)
	65–74 years	58.1	58.1	58.6	57.7
	≥75 years	41.9	41.9	41.4	42.3
	Missing	0.0			
Years of residence in the community		33.6 (18.5)	33.6 (18.5)	33.7 (19.4)	33.6 (17.7)
	Missing	3.1			
Marital status	Married	62.2	65.3	77.1	55.7
	Missing	5.5			
Household composition	Living alone	21.2	21.8	17.8	25.1
	Missing	3.5			
Employment status	Currently working	35.6	37.8	43.2	33.4
	Missing	5.5			
Years of education	≥13 years	19.6	22.8	29.5	17.4
	10–12 years	34.6	40.4	35.6	44.3
	≤9 years	32.4	36.8	34.9	38.4
	Missing	13.3			
Subjective financial stability	Affluent	10.3	11.0	11.0	11.0
	Normal	49.9	52.3	50.9	53.5
	Poor	33.0	36.7	38.1	35.5
	Missing	6.7			
Smoking habit	Yes	12.5	13.5	21.3	7.0
	Missing	5.5			
Regular exercise habit	Yes	53.9	56.9	57.4	56.6
	Missing	6.2			
Body mass index	Underweight (<18.5 kg/m^2^)	6.9	7.6	5.4	9.4
	Normal weight (18.5–24.9 kg/m^2^)	64.7	68.7	69.4	68.1
	Overweight (≥25.0 kg/m^2^)	22.6	23.8	25.3	22.5
	Missing	5.8			
Self-rated health	Good	75.4	79.8	78.9	80.5
	Missing	5.8			
Hypertension	Yes	45.8	45.8	46.6	45.2
	Missing	0.0			
Hyperlipidemia	Yes	12.2	12.2	9.4	14.5
	Missing	0.0			
Heart disease	Yes	11.0	11.0	13.9	8.6
	Missing	0.0			
Stroke	Yes	4.1	4.1	5.8	2.8
	Missing	0.0			
Diabetes	Yes	14.5	14.5	18.1	11.6
	Missing	0.0			
Cancer	Yes	9.3	9.3	10.9	8.1
	Missing	0.0			
Depression	Yes	18.6	20.0	19.6	20.3
	Missing	4.6			
Neighborhood cohesion	Weak	32.4	36.8	40.5	33.7
	Moderate	38.9	42.5	41.8	43.0
	Strong	19.2	20.8	17.7	23.3
	Missing	9.5			
Neighborhood network	Weak	32.1	34.9	48.9	23.5
	Moderate	45.3	49.1	43.0	54.1
	Strong	14.8	16.0	8.2	22.5
	Missing	7.9			
Mortality (until April 2020)		9.0	9.0	13.2	5.6
	Missing	0.0			

Values represent mean (standard deviation) or %.

**Table 2.** Characteristics of the study district

	Mean (SD)	Min–Max
Demographic		
Population (persons)	2,626.7 (1,184.3)	327–7,914
% people aged ≥65 years	22.0 (5.7)	9.5–40.0
% people who have lived in their current neighborhood since birth	7.7 (2.1)	3.5–13.1
Socioeconomic status		
% people who graduated from junior high school only	13.5 (4.5)	4.6–27.0
% people with blue-collar jobs	29.3 (7.4)	9.5–50.5
Cognitive social capital		
% people who felt moderate/strong neighborhood cohesion	63.5 (5.0)	47.7–75.1
Structural social capital		
% people who felt moderate/strong neighborhood network	63.5 (5.8)	45.6–80.0

SD, standard deviation.

The results of the multilevel survival analysis for men are shown in Table 3. In model 1, high district-level neighborhood cohesion (particularly in Q1 and Q2) was associated with a lower risk of mortality. This was consistent after adjusting for individual-level and district-level neighborhood networks in model 3: the HRs were 0.92 (95% CI, 0.84–0.99) for Q1 and 0.91 (95% CI, 0.82–0.99) for Q2, compared to Q5. In contrast, district-level neighborhood networks were not associated with mortality. Table 4 presents the results for women. No association was found between district-level neighborhood cohesion/neighborhood networks and mortality risk.

**Table 3.** Association between district-level social capital and all-cause mortality among men

	Model 1		Model 2		Model 3		Age-interaction model	
	HR	(95% CI)	HR	(95% CI)	HR	(95% CI)	HR	(95% CI)
District-level neighborhood cohesion								
Q1 (highest)	0.93	(0.85–1.02)			0.92	(0.84–0.99)	0.89	(0.80–0.99)
Q2	0.91	(0.82–1.00)			0.91	(0.82–0.99)	0.86	(0.77–0.96)
Q3	0.98	(0.89–1.08)			0.99	(0.89–1.11)	0.93	(0.82–1.05)
Q4	0.95	(0.86–1.06)			0.98	(0.86–1.10)	0.94	(0.82–1.08)
Q5 (lowest)	1.00				1.00		1.00	
District-level neighborhood network								
Q1 (highest)			1.02	(0.93–1.12)	1.03	(0.94–1.14)	1.05	(0.94–1.17)
Q2			1.02	(0.92–1.13)	1.03	(0.92–1.15)	1.11	(0.98–1.25)
Q3			0.99	(0.88–1.10)	1.00	(0.88–1.14)	1.03	(0.90–1.19)
Q4			0.95	(0.84–1.08)	0.95	(0.82–1.10)	0.96	(0.82–1.13)
Q5 (lowest)			1.00		1.00		1.00	
Cross-level interaction								
District-level neighborhood cohesion × age								
Q1 (highest)							1.23	(1.05–1.44)
Q2							1.18	(1.02–1.37)
Q3							1.13	(0.98–1.31)
Q4							1.11	(0.94–1.32)
Q5 (lowest)							1.00	
District-level neighborhood network × age								
Q1 (highest)							0.97	(0.84–1.12)
Q2							0.82	(0.70–0.95)
Q3							0.92	(0.78–1.08)
Q4							0.98	(0.82–1.17)
Q5 (lowest)							1.00	

CI, confidence interval; HR, hazard ratio.

Adjusted for individual-level covariates (age, years of residence in the neighborhood, marital status, household composition, employment status, years of education, subjective financial stability, smoking habit, regular exercise habit, body mass index, self-rated health, hypertension, hyperlipidemia, heart disease, stroke, diabetes, cancer, depression, neighborhood cohesion, and neighborhood network) and district-level covariates (% people aged ≥65 years, % people who have lived in their current neighborhood since birth, % people who graduated from junior high school only, and % people with blue-collar jobs).

**Table 4.** Association between district-level social capital and all-cause mortality among women

	Model 1		Model 2		Model 3		Age-interaction model	
	HR	(95% CI)	HR	(95% CI)	HR	(95% CI)	HR	(95% CI)
District-level neighborhood cohesion								
Q1 (highest)	1.01	(0.88–1.15)			1.01	(0.88–1.16)	1.06	(0.91–1.24)
Q2	0.93	(0.81–1.07)			0.94	(0.81–1.08)	0.95	(0.80–1.11)
Q3	0.94	(0.81–1.08)			0.96	(0.82–1.12)	0.90	(0.75–1.09)
Q4	0.98	(0.84–1.14)			1.01	(0.85–1.20)	0.95	(0.78–1.16)
Q5 (lowest)	1.00				1.00		1.00	
District-level neighborhood network								
Q1 (highest)			1.07	(0.93–1.22)	1.07	(0.93–1.23)	1.00	(0.85–1.17)
Q2			1.06	(0.92–1.23)	1.07	(0.91–1.25)	1.03	(0.86–1.23)
Q3			0.95	(0.81–1.12)	0.96	(0.80–1.15)	0.96	(0.79–1.17)
Q4			0.95	(0.80–1.14)	0.96	(0.78–1.17)	0.91	(0.73–1.15)
Q5 (lowest)			1.00		1.00		1.00	
Cross-level interaction								
District-level neighborhood cohesion × age								
Q1 (highest)							0.89	(0.73–1.08)
Q2							0.97	(0.80–1.19)
Q3							1.13	(0.91–1.40)
Q4							1.15	(0.92–1.45)
Q5 (lowest)							1.00	
District-level neighborhood network × age								
Q1 (highest)							1.18	(0.97–1.43)
Q2							1.09	(0.88–1.34)
Q3							1.00	(0.80–1.24)
Q4							1.13	(0.89–1.43)
Q5 (lowest)							1.00	

CI, confidence interval; HR, hazard ratio.

Adjusted for individual-level covariates (age, years of residence in the neighborhood, marital status, household composition, employment status, years of education, subjective financial stability, smoking habit, regular exercise habit, body mass index, self-rated health, hypertension, hyperlipidemia, heart disease, stroke, diabetes, cancer, depression, neighborhood cohesion [except Model 2], and neighborhood network [except Model 1]) and district-level covariates (% people aged ≥65 years, % people who have lived in their current neighborhood since birth, % people who graduated from junior high school only, and % people with blue-collar jobs).

As a sensitivity analysis, we performed the analysis excluding those who died within a year from the baseline survey to rule out the possibility of reverse causality. The estimates were slightly attenuated but the association between high district-level neighborhood cohesion and lower mortality was still observed (eTable 2).

We examined the cross-level interaction between district-level social capital and individual-level sociodemographic factors (ie, age, years of residence in the neighborhood, marital status, household composition, employment status, years of education, and subjective financial stability). We found a significant interaction between district-level neighborhood cohesion (Q1 and Q2) and age among men (Table 3), but not among women (Table 4). Other cross-level interactions were not statistically significant (data not shown).

Finally, Table 5 shows the results of the sex- and age-stratified analyses. The estimates were predominant in men aged 65–74 years. In particular, the HRs for Q1 and Q2 were 0.83 (95% CI, 0.71–0.97) and 0.82 (95% CI, 0.70–0.97), respectively. In contrast, among men aged ≥75 years, no association between district-level neighborhood cohesion and mortality was observed.

**Table 5.** Age-stratified association between district-level social capital and all-cause mortality among men and women

	Men				Women			
	
	65–74 years		≥75 years		65–74 years		≥75 years	
	HR	(95% CI)	HR	(95% CI)	HR	(95% CI)	HR	(95% CI)
District-level neighborhood cohesion								
Q1 (highest)	0.83	(0.71–0.97)	0.98	(0.87–1.11)	1.19	(0.95–1.49)	0.94	(0.80–1.11)
Q2	0.82	(0.70–0.97)	0.95	(0.83–1.08)	1.03	(0.81–1.31)	0.93	(0.79–1.11)
Q3	0.93	(0.78–1.12)	1.03	(0.90–1.19)	0.95	(0.72–1.25)	0.97	(0.81–1.17)
Q4	0.95	(0.78–1.16)	0.96	(0.82–1.12)	0.97	(0.72–1.32)	1.03	(0.84–1.27)
Q5 (lowest)	1.00		1.00		1.00		1.00	
District-level neighborhood network								
Q1 (highest)	1.11	(0.94–1.30)	1.01	(0.89–1.14)	0.99	(0.78–1.24)	1.11	(0.94–1.31)
Q2	1.25	(0.98–1.48)	0.94	(0.81–1.08)	0.90	(0.68–1.17)	1.17	(0.97–1.41)
Q3	1.11	(0.90–1.36)	0.92	(0.79–1.08)	0.84	(0.62–1.13)	1.00	(0.81–1.24)
Q4	1.10	(0.86–1.40)	0.88	(0.74–1.06)	0.77	(0.54–1.09)	1.05	(0.82–1.34)
Q5 (lowest)	1.00		1.00		1.00		1.00	

CI, confidence interval; HR, hazard ratio.

Adjusted for individual-level covariates (years of residence in the neighborhood, marital status, household composition, employment status, years of education, subjective financial stability, smoking habit, regular exercise habit, body mass index, self-rated health, hypertension, hyperlipidemia, heart disease, stroke, diabetes, cancer, depression, neighborhood cohesion, and neighborhood network) and district-level covariates (% people aged ≥65 years, % people who have lived in their current neighborhood since birth, % people who graduated from junior high school only, and % people with blue-collar jobs).

## DISCUSSION

This study examined the association between community social capital and all-cause mortality using cohort data of community-dwelling older adults in the Tokyo metropolitan area. Many previous studies examining these variables have been conducted in Western nations. Our focus on older urban residents in Japan offers a critical lens through which to explore the nuances of the effects of social capital on health outcomes, thereby contributing valuable knowledge to the field and informing targeted public health strategies. This investigation not only fills a critical gap in existing research but also elucidates potential pathways and mechanisms that lead to a broader discourse on social capital and public health, particularly in aging societies.

We found that men living in districts with high neighborhood cohesion (Q1 and Q2), measured as a cognitive dimension of social capital, had an 8–9% lower mortality risk than those residing in districts with the lowest neighborhood cohesion (Q5). This association was observed to be independent of sociodemographic factors, health behaviors, health conditions, and residential district sociodemographic backgrounds. There are several possible explanations for this relationship. First, men typically have fewer social networks and support systems than women.^18^^,^^19^ Therefore, men who belong to a highly cohesive community may find it easier to obtain social support from neighbors to cope with daily stress and experience a sense of security,^1^ which contributes to longevity. Second, a cohesive community can bring together people’s opinions and point to a clearer and greater need for certain services or amenities.^26^ Attempts to meet these needs could increase the allocation and maintenance of such resources in neighborhoods. Because men are less likely to seek healthcare services than women,^27^ they could obtain more benefits from these services and amenities in the community, which would positively influence their health behaviors and conditions. Third, prior studies suggest that horizontal relations among members can improve societal efficacy by facilitating coordinated actions.^28^ Moreover, a community that possesses high social capital can exert informal social control, effectively preserving social order or intervening in deviant behaviors and attitudes.^29^ Reportedly, men are more likely to engage in risky behaviors/decisions than are women.^30^ Thus, such risky behaviors and decisions may be monitored and intervened for sooner in the community.

The cross-level interaction and the analysis stratified by sex and age indicated that the protective impact of community social cohesion on mortality was pronounced among young-old men; residing in areas with strong neighborhood cohesion (Q1 and Q2) decreased the mortality risk by 17–18% in this population. This cross-level interaction indicates treatment heterogeneity. In Japan, those aged ≥75 years tend to benefit more from formal care services than those aged 65–74 years. For example, the hospital attendance rate and number of people who received care services under the long-term care insurance system were greater among the old-old than among the young-old.^31^^,^^32^ In 2008, Japan established a medical insurance system for people aged ≥75 years. In this system, most people aged ≥75 years must pay 10% of their medical expenses. In comparison, people aged ≤74 years have to cover 20–30% of their medical expenses under the medical insurance system. This implies that older people have more opportunities to obtain administrative and medical care benefits than younger people. This might result in a relatively lower impact of community social capital on mortality in the old-old than in the young-old. In addition, for many men, the places where they spend their daily lives after retirement shift from the workplace to residential communities. Retirement often leads to a decline in social support due to the loss of social contact with coworkers.^33^ Therefore, a more cohesive community would mitigate the decrease in social support and make people feel more comfortable.

Contrary to men, there was no association between community social capital and mortality among women. Women tend to have rich social networks with others^18^^,^^19^ and could receive benefits from their individual networks. Therefore, they may receive relatively less benefits from community social capital. Another reason could be the low mortality rate observed during the follow-up period among women, which might lead to a failure to detect the association. A longer follow-up would be necessary to confirm this finding.

Community characteristics, such as culture, history, and diversity of residents, affect social capital in the community.^34^^,^^35^ It is, therefore, important to investigate the association between social capital and health in various contexts. A previous prefecture-wide study in Shizuoka, Japan reported no association between community social capital and mortality,^16^ while the current study, which was conducted in one ward, found an association, particularly in men. This difference could be attributed to the homogeneity and heterogeneity of the community. Social capital sometimes has different effects and meanings in different contexts, based on the study setting^36^^–^^40^ and individuals’ backgrounds,^21^ even if the same dimension of social capital is measured and analyzed. For example, social capital has a greater effect on health in countries with higher inequality.^40^ The aforementioned Japanese study included multiple local cities and towns in Shizuoka Prefecture^16^; thus, the effect of social capital might be underestimated because its impact varies by area. By contrast, as this study was conducted in a single ward in the Tokyo metropolitan area, the effect of community social capital on mortality was clearly observed.

A meta-analysis indicated that, although social capital is significantly related to a variety of desirable health statuses, the effect sizes are consistently small, regardless of the type of social capital and health outcomes.^41^ Indeed, our results showed a small effect on mortality in men (8–9%). Fostering community social capital can be regarded as a way of the population approach. The total benefit from the population approach can be large even though each individual receives only a small benefit.^42^ Thus, although small, the impact of district-level neighborhood cohesion would be significant. Community social capital might not naturally increase; to foster the same, some “device” (ie, intervention) would be necessary within the community. Various approaches to enhancing community social capital for improving health outcomes have been suggested.^43^ In Japan, intergenerational programs involving children and older generations have been shown to strengthen neighborhood trust.^44^ Policymakers should consider these interventions and apply optimal ones to health policy, as the effectiveness of social capital interventions depends on specific circumstances.

This study had several limitations. First, there is a possibility of selection bias due to the response rate (approximately 60%). People with severe diseases or disabilities were excluded from the baseline survey. Therefore, our results may have underestimated the association between community social capital and mortality. Second, district-level social capital variables were created by aggregating the individual responses of the study participants within each district. Non-participants in the survey and young and middle-aged people living in the district were excluded. To develop more genuine and reflective contextual indicators of reality, these conditions should be considered. Third, the target community was limited to Adachi Ward. Therefore, care should be taken when generalizing these findings.

In conclusion, this study highlights that higher district-level neighborhood cohesion is associated with a lower risk of mortality in community-dwelling older men in an urban area of Japan, particularly among those aged 65–74 years. We demonstrated a clear survival advantage for older adults living in a cohesive community, supporting the notion that interventions aimed at enhancing community social capital could be a strategic component of public health efforts to reduce mortality. Our findings suggest that policymakers should focus on fostering social cohesion in the community as a strategy for healthy aging in addition to developing approaches that target individuals. More research on the relationship between community social capital and all-cause mortality should be conducted, such as in rural areas or in different Asian settings.

## SUPPLEMENTARY MATERIAL

eTable 1. Literature review of research focusing on the relationship between community social capital and all-cause mortality

eTable 2. Association between district-level social capital and all-cause mortality after excluding those who died within a year after the baseline survey in men and women

## References

[1] Kawachi I, Berkman LF. Social capital, social cohesion, and health. In: Berkman LF, Kawachi I, Glymour MM, eds. *Social Epidemiology*. New York: Oxford University Press; 2014:290–319.

[2] Ehsan A, Klaas HS, Bastianen A, Spini D. Social capital and health: a systematic review of systematic reviews. SSM Popul Health. 2019;8:100425. 10.1016/j.ssmph.2019.100425 31431915 PMC6580321

[3] Meijer M, Röhl J, Bloomfield K, Grittner U. Do neighborhoods affect individual mortality? A systematic review and meta-analysis of multilevel studies. Soc Sci Med. 2012;74:1204–1212. 10.1016/j.socscimed.2011.11.034 22365939

[4] Murayama H, Fujiwara Y, Kawachi I. Social capital and health: a review of prospective multilevel studies. J Epidemiol. 2012;22:179–187. 10.2188/jea.JE20110128 22447212 PMC3798618

[5] Kawachi I, Kennedy BP, Lochner K, Prothrow-Stith D. Social capital, income inequality, and mortality. Am J Public Health. 1997;87:1491–1498. 10.2105/AJPH.87.9.1491 9314802 PMC1380975

[6] Lochner KA, Kawachi I, Brennan RT, Buka SL. Social capital and neighborhood mortality rates in Chicago. Soc Sci Med. 2003;56:1797–1805. 10.1016/S0277-9536(02)00177-6 12639596

[7] Kennelly B, O’Shea E, Garvey E. Social capital, life expectancy and mortality: a cross-national examination. Soc Sci Med. 2003;56:2367–2377. 10.1016/S0277-9536(02)00241-1 12742601

[8] Giordano GN, Mewes J, Miething A. Trust and all-cause mortality: a multilevel study of US General Social Survey data (1978–2010). J Epidemiol Community Health. 2019;73:50–55. 10.1136/jech-2018-211250 30322881 PMC6839792

[9] Wen M, Cagney KA, Christakis NA. Effect of specific aspects of community social environment on the mortality of individuals diagnosed with serious illness. Soc Sci Med. 2005;61:1119–1134. 10.1016/j.socscimed.2005.01.026 15970225

[10] Mohan J, Twigg L, Barnard S, Jones K. Social capital, geography and health: a small-area analysis for England. Soc Sci Med. 2005;60:1267–1283. 10.1016/j.socscimed.2004.06.050 15626523

[11] Sundquist K, Hamano T, Li X, Kawakami N, Shiwaku K, Sundquist J. Linking social capital and mortality in the elderly: a Swedish national cohort study. Exp Gerontol. 2014;55:29–36. 10.1016/j.exger.2014.03.007 24632181

[12] Islam MK, Gerdtham UG, Gullberg B, Lindström M, Merlo J. Social capital externalities and mortality in Sweden. Econ Hum Biol. 2008;6:19–42. 10.1016/j.ehb.2007.09.004 18280227

[13] Pattussi MP, Anselmo Olinto MT, Rower HB, Souza de Bairros F, Kawachi I. Individual and neighbourhood social capital and all-cause mortality in Brazilian adults: a prospective multilevel study. Public Health. 2016;134:3–11. 10.1016/j.puhe.2015.12.007 26809862

[14] Choi S, Oh J, Park SM, . Association of community level social trust and reciprocity with mortality: a retrospective cohort study. BMC Public Health. 2020;20:1793. 10.1186/s12889-020-09944-3 33239007 PMC7690021

[15] Blakely T, Atkinson J, Ivory V, Collings S, Wilton J, Howden-Chapman P. No association of neighbourhood volunteerism with mortality in New Zealand: a national multilevel cohort study. Int J Epidemiol. 2006;35:981–989. 10.1093/ije/dyl088 16931531

[16] Inoue S, Yorifuji T, Takao S, Doi H, Kawachi I. Social cohesion and mortality: a survival analysis of older adults in Japan. Am J Public Health. 2013;103:e60–e66. 10.2105/AJPH.2013.301311 24134379 PMC3828960

[17] Oskarsson S, Dawes C, Johannesson M, Magnusson PKE. The genetic origins of the relationship between psychological traits and social trust. Twin Res Hum Genet. 2012;15:21–33. 10.1375/twin.15.1.21 22784450

[18] Antonucci TC, Akiyama H. An examination of sex differences in social support among older men and women. Sex Roles. 1987;17:737–749. 10.1007/BF00287685

[19] McLaughlin D, Vagenas D, Pachana NA, Begum N, Dobson A. Gender differences in social network size and satisfaction in adults in their 70s. J Health Psychol. 2010;15:671–679. 10.1177/1359105310368177 20603290

[20] OECD. *Health at a Glance 2023: OECD Indicators*. Paris: OECD Publishing; 2023.

[21] Murayama H, Nishi M, Nofuji Y, . Longitudinal association between neighborhood cohesion and depressive mood in old age: a Japanese prospective study. Health Place. 2015;34:270–278. 10.1016/j.healthplace.2015.05.015 26113436

[22] Murayama H, Sugiyama M, Inagaki H, . Is community social capital associated with subjective symptoms of dementia among older people? A cross-sectional study in Japan. Geriatr Gerontol Int. 2018;18:1537–1542. 10.1111/ggi.13519 30184611

[23] Kawachi I, Subramanian SV, Kim D. Social capital and health: a decade of progress and beyond. In: Kawachi I, Subramanian SV, Kim D, eds. *Social Capital and Health*. New York: Springer; 2008:1–26.

[24] Whooley MA, Avins AL, Miranda J, Browner WS. Case-finding instruments for depression. Two questions are as good as many. J Gen Intern Med. 1997;12:439–445. 10.1046/j.1525-1497.1997.00076.x 9229283 PMC1497134

[25] Oakes JM. The (mis)estimation of neighborhood effects: causal inference for a practicable social epidemiology. Soc Sci Med. 2004;58:1929–1952. 10.1016/j.socscimed.2003.08.004 15020009

[26] Altschuler A, Somkin CP, Adler NE. Local services and amenities, neighborhood social capital, and health. Soc Sci Med. 2004;59:1219–1229. 10.1016/j.socscimed.2004.01.008 15210093

[27] Milner A, Disney G, Byars S, King TL, Kavanagh AM, Aitken Z. The effect of gender on mental health service use: an examination of mediation through material, social and health-related pathways. Soc Psychiatry Psychiatr Epidemiol. 2020;55:1311–1321. 10.1007/s00127-020-01844-6 32055895

[28] Putnam RD. *Making Democracy Work: Civic Traditions in Modern Italy*. New Jersey: Princeton University Press; 1993.

[29] Sampson RJ, Raudenbush SW, Earls F. Neighborhoods and violent crime: a multilevel study of collective efficacy. Science. 1997;277:918–924. 10.1126/science.277.5328.918 9252316

[30] Byrnes JP, Miller DC. Gender differences in risk taking: a meta-analysis. Psychol Bull. 1999;125:367–383. 10.1037/0033-2909.125.3.367

[31] Cabinet Office. *White Paper on Aging Society 2022*. Tokyo: Sanwa; 2022.

[32] Ministry of Health Lobour, and Welfare. Comprehensive Survey of Living Conditions 2022. 2022. https://www.mhlw.go.jp/toukei/saikin/hw/k-tyosa/k-tyosa22/index.html (accessed on April 1, 2024).

[33] Bossé R, Aldwin CM, Levenson MR, Spiro A III, Mroczek DK. Change in social support after retirement: longitudinal findings from the Normative Aging Study. J Gerontol. 1993;48:P210–P217. 10.1093/geronj/48.4.P210 8315238

[34] Hanibuchi T, Kondo K, Nakaya T, Shirai K, Hirai H, Kawachi I. Does walkable mean sociable? Neighborhood determinants of social capital among older adults in Japan. Health Place. 2012;18:229–239. 10.1016/j.healthplace.2011.09.015 22000345

[35] Murayama H, Arami R, Wakui T, Sugawara I, Yoshie S. Cross-level interaction between individual and neighbourhood socioeconomic status in relation to social trust in a Japanese community. Urban Stud. 2014;51:2770–2786. 10.1177/0042098013513648

[36] Tsubokawa T, Shobugawa Y, Iguchi S, . Do community social capital and built environment associate with homebound in older adults? The JAGES Niigata Study. J Epidemiol. 2022;32:254–269. 10.2188/jea.JE20200154 34121046 PMC9086311

[37] Yamamura E. Comparison of social trust’s effect on suicide ideation between urban and non-urban areas: the case of Japanese adults in 2006. Soc Sci Med. 2015;140:118–126. 10.1016/j.socscimed.2015.07.001 26218455

[38] Han Y, Chung RY. The role of neighborhood social capital on health and health inequality in rural and urban China. Prev Med. 2022;156:106989. 10.1016/j.ypmed.2022.106989 35150751

[39] Ziersch AM, Baum F, Darmawan IG, Kavanagh AM, Bentley RJ. Social capital and health in rural and urban communities in South Australia. Aust N Z J Public Health. 2009;33:7–16. 10.1111/j.1753-6405.2009.00332.x 19236353

[40] Islam MK, Merlo J, Kawachi I, Lindström M, Burström K, Gerdtham UG. Does it really matter where you live? A panel data multilevel analysis of Swedish municipality-level social capital on individual health-related quality of life. Health Econ Policy Law. 2006;1:209–235. 10.1017/S174413310600301X 18634694

[41] Xue X, Reed WR, Menclova A. Social capital and health: a meta-analysis. J Health Econ. 2020;72:102317. 10.1016/j.jhealeco.2020.102317 32497954

[42] Rose G. *The Strategy of Preventive Medicine*. New York: Oxford University Press; 1992.

[43] Villalonga-Olives E, Wind TR, Kawachi I. Social capital interventions in public health: a systematic review. Soc Sci Med. 2018;212:203–218. 10.1016/j.socscimed.2018.07.022 30048843

[44] Murayama Y, Murayama H, Hasebe M, Yamaguchi J, Fujiwara Y. The impact of intergenerational programs on social capital in Japan: a randomized population-based cross-sectional study. BMC Public Health. 2019;19:156. 10.1186/s12889-019-6480-3 30727981 PMC6364465

