# Supplementary material for: Community Social Capital and All-cause Mortality in Japan: Findings From the Adachi Cohort Study
Source: J Epidemiol. 2025 Jun 5;35(6):270–7. doi: 10.2188/jea.JE20240277 (PMC12066192; doi:10.2188/jea.JE20240277)
Supplement: Supplementary file 1 [file je-35-270-s001.pdf]

eTable 1. Literature review of research focusing on the relationship between community social capital and all-cause mortality

Authors (year)	Study area (country)	Study design	Social capital dimension		Summary
			Cognitive	Structural	
Kawachi et al. (1997)[5]	United States (nationwide)	Ecological	X	X	Both greater social trust and group membership were associated with lower all-cause mortality at the state level.
Lochner et al. (2003)[6]	Chicago, United States	Ecological	X	X	Neighborhood higher social capital (measured by reciprocity, trust, and civic participation) was associated with lower neighborhood death rates.
Kennelly et al. (2003)[7]	19 countries in the Organization for Economic Co-operation and Development (OECD)	Ecological	X	X	Indicators of social capital (trust and membership in voluntary associations) were not associated with life expectancy at the country level.
Giordano et al. (2019)[8]	United States (nationwide)	Multilevel	X		Region-level higher generalized trust was associated with lower all-cause mortality.
Wen et al. (2005)[9]	Chicago, United States	Multilevel	X	X	Higher collective efficacy at the zip code area level was associated with lower mortality. Community social network density (measured by the size of social network and frequency of social interaction) was not protective but detrimental.
Mohan et al. (2005)[10]	England, Wales, and Scotland (nationwide)	Multilevel	X	X	Lower proportions of several activities (e.g., voluntary activity, social activity, altruistic activity, and political activity) in the area were associated with higher mortality.

Sundquist et al. (2014)[11]	Sweden (nationwide)	Multilevel		X	Neighborhood-level higher linking social capital (measured by voting rate) was associated with lower all-cause mortality.
Islam et al. (2008)[12]	Sweden (nationwide)	Multilevel		X	Municipality-level social capital indicators (registered election participation rate and registered crime rate) were associated with individual risk from all-cause mortality for male participants older than 65 years but not for female participants.
Pattussi et al. (2016)[13]	Municipality of São Leopoldo, Brazil	Multilevel	X		Neighborhood-level low social action was associated with higher mortality.
Choi et al. (2020)[14]	South Korea (nationwide)	Multilevel	X		Higher social trust and reciprocity at the district level were associated with lower all-cause mortality.
Blakely et al. (2006)[15]	New Zealand (nationwide)	Multilevel		X	There was no significant association between neighborhood-level volunteerism and all-cause mortality.
Inoue et al. (2012)[16]	Shizuoka prefecture, Japan	Multilevel	X		No statistically significant relationship was found between district-level social cohesion and mortality risk.

eTable 2. Association between district-level social capital and all-cause mortality after excluding those who died within a year after the baseline survey in men and women

	Men		Women	
	HR	(95% CI)	HR	(95% CI)
District-level neighborhood cohesion				
Q1 (highest)	0.94	(0.88–1.00)	1.01	(0.87–1.17)
Q2	0.93	(0.86–0.99)	0.93	(0.80–1.09)
Q3	1.03	(0.92–1.17)	0.99	(0.84–1.17)
Q4	1.02	(0.90–1.17)	1.03	(0.86–1.24)
Q5 (lowest)	1.00		1.00	
District-level neighborhood network				
Q1 (highest)	1.01	(0.91–1.13)	1.08	(0.93–1.25)
Q2	1.01	(0.90–1.14)	1.06	(0.89–1.25)
Q3	0.97	(0.84–1.11)	0.96	(0.79–1.17)
Q4	0.92	(0.78–1.07)	0.94	(0.75–1.17)
Q5 (lowest)	1.00		1.00	

CI, confidence interval; HR, hazard ratio.

Adjusted for individual-level covariates (age, years of residence in the neighborhood, marital status, household composition, employment status, years of education, subjective financial stability, smoking habit, regular exercise habit, body mass index, self-rated health, hypertension, hyperlipidemia, heart disease, stroke, diabetes, cancer, depression, neighborhood cohesion, and neighborhood network) and district-level covariates (% people aged ≥65 years, % people who have lived in their current neighborhood since birth, % people who graduated from junior high school only, and % people with blue-collar jobs).
